# Foliar Roughness and Water Content Impact on *Escherichia coli* Attachment in Baby Leafy Greens

**DOI:** 10.3390/biology12010102

**Published:** 2023-01-09

**Authors:** Stefania Truschi, Ada Baldi, Piero Bruschi, Ilaria Cacciari, Massimiliano Marvasi, Anna Lenzi

**Affiliations:** 1 Department of Agriculture, Food, Environment and Forestry (DAGRI), University of Florence, 50144 Florence, Italy; 2 CNR, Institute of Applied Physics ‘Nello Carrara’, Sesto Fiorentino, 50019 Florence, Italy; 3 Department of Biology, University of Florence, Sesto Fiorentino, 50019 Florence, Italy

**Keywords:** food-borne diseases, enterobacteria, leafy vegetables, stomata, leaf roughness, foliar water content, UV treatment

## Abstract

**Simple Summary:**

Vegetables may host human pathogens, such as *Salmonella enterica* and *Escherichia coli*. Therefore, the consumption of salads may raise safety concerns as they are eaten raw and often without prior proper washing. Bringing to light the differences between different salad crops in the susceptibility to human pathogen contamination and understanding the relationship between the susceptibility and leaf traits can contribute to increase food safety. We evaluated the susceptibility to *E. coli* attachment of 30 different baby leaves (leafy vegetables harvested at an early stage of growth and consumed as salads), finding inter- and intraspecific variation. In romaine lettuce, the most involved in food disease outbreaks in the U.S., we found genotypes (‘Maraichere’) less susceptible to contamination than others (‘Bionda degli Ortolani’). Among the 30 baby leaves, ‘Bionda degli Ortolani’, rocket and Swiss chard were the most susceptible, with wild rocket, wild lettuce and lamb’s lettuce the least. Further analysis of these six baby leaves revealed that leaf roughness and water content were positively correlated with attachment level. In rougher leaves, we observed a lower effectiveness of UV treatment in reducing attachment.

**Abstract:**

Understanding the relation between the susceptibility of different leafy greens to human pathogen contamination and leaf traits can contribute to increase the food safety of the fresh vegetable industry. The aim of this research was to evaluate the susceptibility to *E. coli* ATCC 35218 attachment in 30 accessions of baby leaves, and to identify leaf traits potentially involved in the contamination. The accessions were surface inoculated with a bacterial suspension containing 1 × 10^7^ cells/mL and the attachment was measured 1.5 h after inoculation. Significant differences in attachment were detected between the accessions for *p* ≤ 0.05. The three most and the three least susceptible accessions were selected and characterized for leaf micro-morphological traits (stomata density and size, surface roughness) and water content. Scanning electron microscopy was used to analyse the stomatal parameters. Roughness was measured by an innovative portable 3D digital microscope. No significant correlation between the attachment of *E. coli* ATCC 35218 and stomatal parameters was detected, while the attachment was positively correlated with roughness and water content. The *E. coli* ATCC 35218 population in surface-inoculated leaves was also measured after a UV treatment, which was found to be less effective in reducing bacterial contamination in the rougher leaves. This result suggested that roughness offers UV protection, further highlighting its impact on the microbiological safety of baby leafy greens.

## 1. Introduction

Baby leaves are leafy vegetables harvested at an early stage of growth (up to the eighth true leaf) and consumed as salads [1]. They can be commercialized as unprocessed produce but are often minimally processed and sold in a ready-to-eat form, which offers the advantage of ease of consumption. With the increased consumer awareness of the relationship between health and the presence of fresh vegetables in the diet, the demand for minimally processed fruits and vegetables is constantly growing [2]. The global size of the ready-to-eat salad market was valued at USD 10.78 billion in 2020 and is expected to further expand in the next years [3]. 

A high number of species are grown as baby leaf salads. Lettuce, with many types of different colours and shapes, is the most important, but also a lot of other vegetable crops are used, including endive (*Cichorium endivia* L.), chicory (*Cichorium intybus* L.), spinach (*Spinacia oleracea* L.), Swiss chard (*Beta vulgaris* L.), mustard (*Brassica juncea* L.), kale (*Brassica oleracea* L.), rocket (*Eruca sativa* Miller), etc. [1]. Furthermore, wild greens (leafy plants gathered in the wild and used as food) have also been demonstrated to be suitable for producing baby leaves [4]. The different leaves can be marketed individually or as salad mixes.

It is well known that vegetables are prone to host human pathogens, including Enterobacteriaceae [5], with contaminations that may occur through various routes from farm to fork [6]. Salads entail great risks since they are consumed raw, and usually without prior proper washing. For fresh-cut salads, a raising trend in foodborne disease has been observed following the increase in consumption. Fresh produce was reported to be a major vehicle of foodborne diseases in both U.S. and Europe, in most cases with the coliforms *Salmonella enterica* and *Escherichia coli* serotype O157:H7 as causative agents [7]. One of the last food safety alerts reported by the Centers for Disease Control and Prevention (CDC) for the U.S. in 2021 involved a multistate *E. coli* outbreak linked to a packaged salad containing spinach, mizuna, kale and chard baby leaves [8].

Attachment to the plant phyllosphere is the first step in product contamination and can be followed by internalization in the absence of timely decontamination treatments [9,10]. The involvement of leaf traits (e.g., veins, stomata, trichomes, roughness and wettability) in bacterial attachment and further persistence has been demonstrated. These features mainly depend on plant species and cultivars and on leaf age [11,12,13,14,15]. Stomata are one of the preferred sites of bacteria for attachment [16] because they can provide protection, moisture and nutrients for their survival [17] and represent a route of internalization into the plant foliar tissue [18,19]. The presence of bulges and hollows on leaf surface influences bacteria distribution and movement, at the same time increasing the area for the attachment [20,21]. *E. coli* attachment and persistence was found to be positively correlated with stomatal density, stomata size and surface roughness by many authors [11,22,23,24]. It can be hypothesized that also the leaf water content can play a role in the contamination by human pathogens. In fact, Yadav et al. [25] demonstrated that bacterial colonization of the phyllosphere in eight Mediterranean plant species depends on leaf hydration, where leaves with a higher water content are more highly colonized [25].

In this study, *E. coli* ATCC 35218 was used as a model microorganism to evaluate the susceptibility to enterobacteria contamination in 30 accessions of baby leaves belonging to 13 different species. Then, a selected number of accessions (those with the highest and the lowest susceptibility) were characterized for leaf micro-morphological traits (stomata density and size and surface roughness) and water content to identify the leaf traits possibly involved in the bacterial adherence. Information about the susceptibility of different baby leaf accessions to *E. coli* contamination and the related traits can contribute to increasing the food safety of the fresh vegetable industry.

## 2. Materials and Methods

### 2.1. Baby Leaves Cultivation and Characterization

Plants of 30 different accessions belonging to 13 species were tested (Table 1).

After sowing, seeds were kept for 48 h in the dark at 20 °C for promoting germination. Seedlings were hydroponically grown in a floating system in a growth chamber at 21 ± 2 °C (day) and 14 ± 2 °C (night) with a photoperiod of 16 h under fluorescent lighting units OSRAM L36W/77 (36 W, 120 cm in length, 26 mm in diameter) up to the baby-leaf stage (five/six weeks after sowing, depending on the species). A full-strength Hoagland’s nutrient solution N 15.0 mM, *p* 0.10 mM, K 6.0 mM, Ca 5.0 mM, Mg 2.0 mM, Fe 50.0 μM, B 46.2 μM, Mn 9.2 μM, Zn 0.78 μM, Cu 0.32 μM, Mo 0.12 μM) was used. Baby leaves were cut at the base of the petiole, and immediately used for the bacterial attachment assay. The average fresh weight, dry weight, leaf number, and leaf area of the baby leaf plants are reported in Table S1. Fresh weight (FW) and dry weight (DW) were measured before and after oven desiccation at 80 °C for 48 h or until a constant weight, and water content was calculated as [(FW-DW)/FW]*100. Leaf area was determined by a planimeter LI-3000 (LI-COR, Lincoln, NE, USA). Colour was measured with the Handy Colorimeter NR-3000 (Nippon Denshoku Kogyo C., LTD.) and expressed as a*, b* and L* values (Table S2).

### 2.2. Bacterial Strain

*Escherichia coli* ATCC 35218 was used for the inoculation assays. This strain (classified as biosafety level 1) has specific virulence genes associated with different *E. coli* pathotypes related to human and animal infections [26].

### 2.3. Surface Inoculation of the Baby Leaves 

The bacterial inocula was started from a −20 °C glycerol stock. Briefly, one mL from an overnight inoculum in Lysogeny Broth (LB, Oxoid, UK) was washed and diluted in sterile Physiological Solution (PS, NaCl 0.85% w/v in H_2_O) to clean the cells from LB medium [27]. Disks 1.5 cm in diameter were cut from the leaves of the 30 accessions used in this study and put in Petri plates. Fifty µL of the working bacterial suspension (containing approximately 1 × 10^7^ cells/mL) were placed onto the centre of the leaf disk on the adaxial side. Leaves were incubated for 1.5 h at 25 °C in static. After incubation, the disks were washed three times in 30 mL of sterile PS in Petri plates to remove unattached bacterial cells. The leaf disks were placed on the adaxial side on the first Petri plate and gently washed by manually rotating the plates clockwise 30 times; then, the same procedure was repeated on the second and the third plate. Rinsed disks were grinded with a mini-pestle (into 1.5 mL tubes) in 0.5 mL of PS. After grinding, 20 µL were plated onto selective and differential media MacConkey Agar (Oxoid, UK) and incubated at 37 °C overnight. Colony-forming units (CFU) were counted and log base 10 (log) transformed. Two different seeding sets and the third true leaf of 5 plants at the baby leaf stage from the same seeding set were used for each accession (10 leaves in total/accession). 

Among the 30 accessions, we selected the three most and three least contaminated. 

### 2.4. Analysis of Leaf Micro-Morphological Traits 

Stomatal parameters and roughness were determined in the six selected accessions.

#### 2.4.1. Stomata

Scanning electron microscopy (SEM) was used to analyse the stomata density and size (length, width and stomatal rim area) of the adaxial leaf surface. Before SEM observations, fresh leaf samples were coated by a thin layer of gold (~10 nm) to reduce shrinkage while ensuring the preservation of cell structures as close to the natural state as possible [28]. Eight observations (the third true leaf of 4 plants at the baby leaf stage and 2 sections per leaf) were performed in each accession. Photographs at 1 × 300 and 1 × 600 were used for counting stomata and measuring stomata sizes, respectively. Stomata were counted on the images (view field ranging from 923 to 929 μm^2^), and then the number per surface unit (mm^2^) was calculated by a simple proportion. ImageJ software [29] was used to measure stomata size; the measure was expressed in μm by means of a proportion considering the bar of known length present in each image.

#### 2.4.2. Roughness

A portable prototype of 3D digital microscope was used to measure the leaf surface roughness [30]. Basically, its setup includes three elements: an optoelectronic imaging group (a CCD digital camera and a fixed focus optical element), a calibrated stage for translating this group along the optical axis, and a smart polymeric lightning system [31]. The 3D reconstruction of the surface under inspection was obtained by elaborating a sequence of defocused images (Video S1) and implementing a suitable algorithm based on shape from a focusing technique [32]. Surface roughness parameters were extracted from 3D reconstruction, according to ISO standards [33]. In particular, we focused the analysis of plant surface on the average roughness (Ra) and root mean square roughness (Rq).

A suitable optical element was used in order to achieve a field of view of about 7 × 5.2 mm and a vertical resolution of about 10 µm. It is worth noting that these kinds of measurements are completely non-contact, as well as non-destructive; this overcomes the disadvantages of using a contact stylus profilometer with soft biological samples. Five different analyses in two leaves (10 replicates) were performed for each accession.

### 2.5. UV Treatment

The leaves of the six selected accessions were surface-inoculated as described above (5 leaf disks per accession) and then exposed for 5 min under a UV lamp (UVC ≥ 90% with 108.4 μW/cm^2^ from 0.5 m) placed at a distance of 10 cm. After exposure, CFU were counted and log transformed as mentioned above. As a control, three separate drops of 50 µL of 10^7^ *E. coli* cell/mL inoculum were put onto a smooth plastic surface and exposed to the above described UV treatment. The effectiveness of the treatment on the control was demonstrated by complete bacterial inactivation after plating onto MacConkey Agar. 

### 2.6. Statistical Analysis

Data were subjected to the Shapiro–Wilk test for normality and Levene’s test for homogeneity of variances. Analysis of variance (ANOVA) was performed using the CoStat software (version 6.45, Monterey, CA, USA). Significance was set at *p* ≤ 0.05 (Tukey test). The Pearson’s correlation test (*p* ≤ 0.05) was used to determine the relationship between the *E. coli* ATCC 35218 attachment and all the considered leaf traits.

## 3. Results

### 3.1. E. coli Attachment in the Baby Leaves

Variability in *E. coli* ATCC 35218 attachment was observed among the 30 studied baby leaf accessions, even within the same species (Table 2). The romaine lettuce ‘Bionda degli Ortolani’ showed the highest contamination, with significant differences compared to wild chicory (local accession), romaine lettuce ‘Maraichere’, lettuce ‘Pamela’, sorrel, wild rocket ‘Yeti’, wild lettuce and lamb’s lettuce ‘Trophy F1′ (*p* < 0.001). The latter resulted in being less susceptible than all the other accessions. Considering the data by species, differences were found not only in *Lactuca sativa,* but also in *Diplotaxis tenuifolia*; conversely, no differences were observed within *Taraxacum campylodes*, *Brassica juncea, Brassica rapa*, *Cichorium intybus* and *Beta vulgaris*. Among the lettuces, ‘Bionda degli Ortolani’ showed a contamination level of 3.36 ± 0.36 log CFU/cm^2^, significantly different from ‘Maraichere’ (2.65 ± 0.34 log CFU/cm^2^) and ‘Pamela’ (2.45 ± 0.20 log CFU/cm^2^). In *Diplotaxis tenuifolia*, ‘Yeti’ was less susceptible than the Ingegnoli accession.

**Figure 1:**
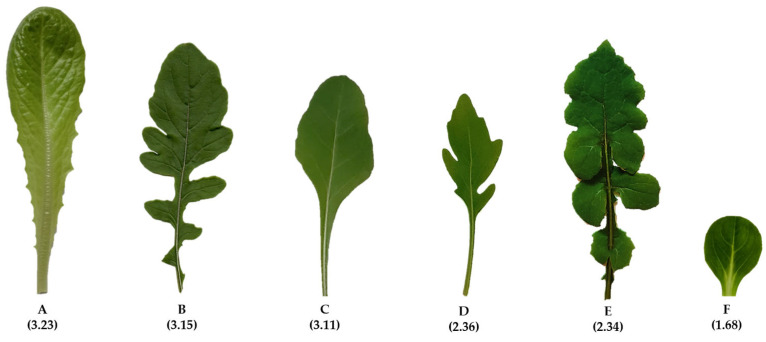
The six selected accessions: romaine lettuce ‘Bionda degli Ortolani’ (**A**), Swiss chard (**B**), and rocket (**C**) as the most susceptible to contamination, and wild rocket ‘Yeti’ (**D**), wild lettuce (**E**), and lamb’s lettuce ‘Trophy F1′ (**F**) as the least susceptible. Attachment values in brackets (log CFU/cm^2^).

Based on the results of the attachment assays, we selected six accessions (romaine lettuce ‘Bionda degli Ortolani’, Swiss chard, and rocket as the most susceptible, and wild rocket ‘Yeti’, wild lettuce, and lamb’s lettuce ‘Trophy F1′ as the least susceptible; Figure 1). Further analyses aimed to identify traits possibly involved in the bacterial retention. 

### 3.2. Leaf Micro-Morphological Traits and Water Content

**Figure 2:**
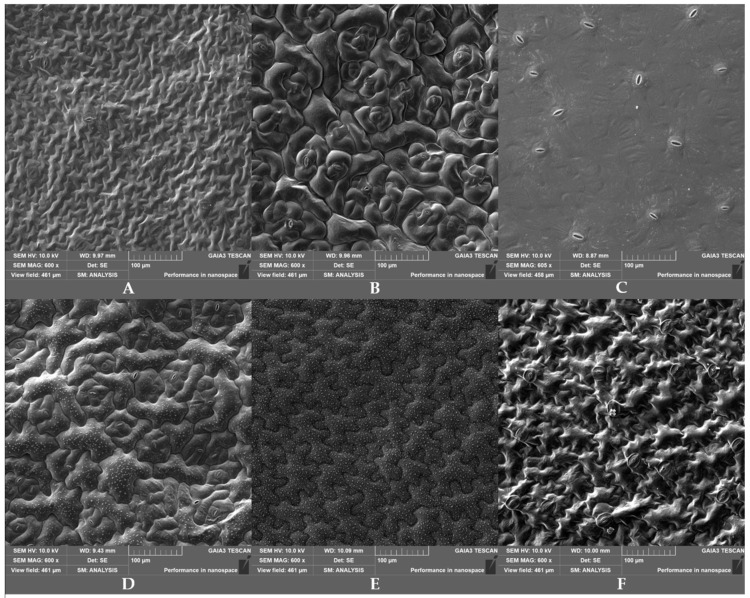
SEM image (600×) of adaxial leaf surface of the six selected accessions: romaine lettuce ‘Bionda degli Ortolani’ (**A**), Swiss chard (**B**), rocket (**C**), wild rocket ‘Yeti’ (**D**), wild lettuce (**E**), and lamb’s lettuce ‘Trophy F1′ (**F**). The absence of stomata can be noticed in wild lettuce.

The SEM images (Figure 2) highlighted significant differences in stomata density and size (length, width and rim area) between the six selected baby leaf accessions (Table 3). In wild lettuce, these parameters were not measured as this species did not have stomata on the adaxial leaf surface (Figure 2E). Some of the observed differences were consistent with the contamination data: e.g., the absence of stomata in wild lettuce, the highest and lowest stomata density in rocket (92.20 ± 28.40) and lamb’s lettuce ‘Trophy F1′ (57.14 ± 5.47), respectively, and the highest stomatal rim area in Swiss chard (45.58 ± 6.78 µm^2^). However, no significant correlation of *E. coli* attachment ATCC 35218 versus any of the stomatal parameters was detected (data not shown).

**Figure 3:**
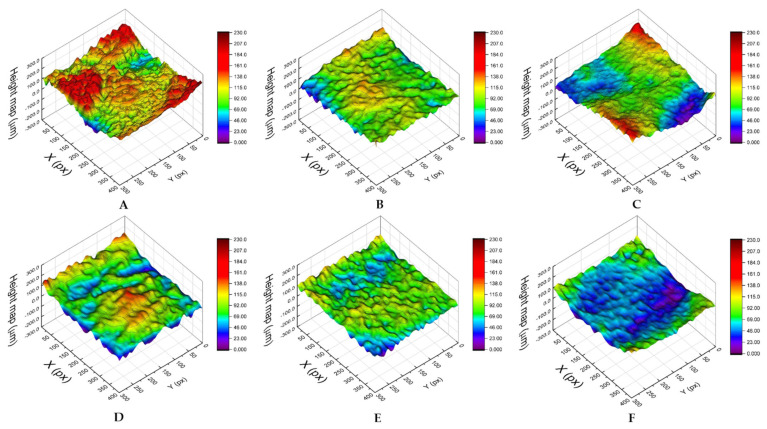
Examples of leaf surface 3D reconstructions of the six selected accessions: romaine lettuce ‘Bionda degli Ortolani’ (**A**), Swiss chard (**B**), rocket (**C**), wild rocket ‘Yeti’ (**D**), wild lettuce (**E**) and lamb’s lettuce ‘Trophy F1′ (**F**). X and Y axes are expressed in pixels (1 pixel = 17.5 µm), with heights reported on the vertical axis (µm). The corresponding color ramp is shown on the right of each reconstruction.

**Figure 4:**
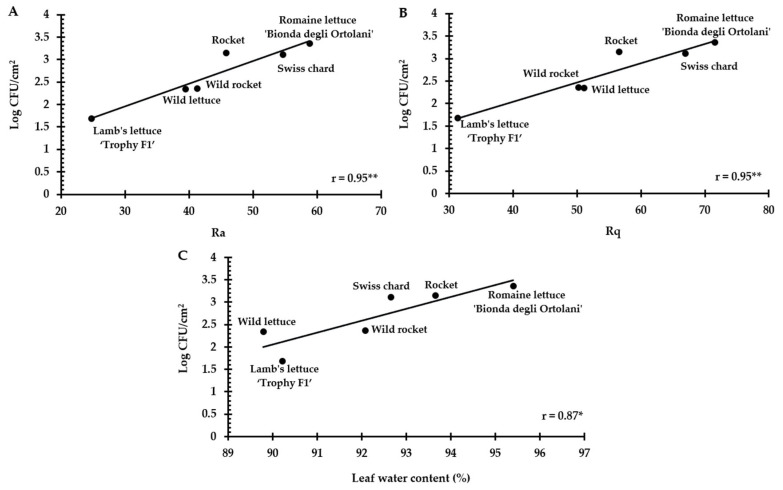
Correlation between *E. coli* ATCC 35218 attachment and (**A**,**B**) roughness (Ra and Rq) and (**C**) leaf water content % in the six selected accessions. * Significant per *p* < 0.05; ** significant per *p* < 0.01.

Three-dimensional digital microscopy revealed significant differences (*p* < 0.001) between the accessions for both the roughness parameters, with romaine lettuce ‘Bionda degli Ortolani’ and Swiss chard (more susceptible accessions) showing higher values (Ra = 58.81 ± 11.52 and 54.70 ± 15.84, respectively; Rq = 71.56 ± 13.71 and 66.92 ± 18.35, respectively) than less susceptible accessions, i.e., wild rocket ‘Yeti’ (Ra = 41.33 ± 4.01; Rq = 50.23 ± 5.01), wild lettuce (Ra = 39.43 ± 12.42; Rq = 51.14 ± 15.49) and lamb’s lettuce ‘Trophy F1′ (Ra = 24.74 ± 10.92; Rq = 31.38 ± 14.24) (Table 3). From a visual point of view, the differences in leaf roughness can be observed in the 3D reconstructions shown in Figure 3. A rough estimate of the Ra parameter can be obtained considering the differences between the maximum and minimum height values, which are higher in ‘Bionda degli Ortolani’ (Figure 3A) and lower in lamb’s lettuce ‘Trophy F1′ (Figure 3F). The Pearson’s test revealed a significant correlation between contamination level and both Ra and Rq values (Figure 4A,B).

Moreover, significant differences (*p* < 0.001) between the selected accessions were observed in leaf water content (Table 3). The accession most susceptible to contamination (‘Bionda degli Ortolani’) showed the highest leaf water content (95.36 ± 0.84%) and differed from all the others, but especially from the least susceptible accessions, i.e., wild lettuce (89.79 ± 1.13%) and lamb’s lettuce ‘Trophy F1′ (90.22 ± 2.30%). The correlation between *E. coli* ATCC 35218 attachment and water content was significant (Figure 4C).

### 3.3. UV Experiment

**Figure 5:**
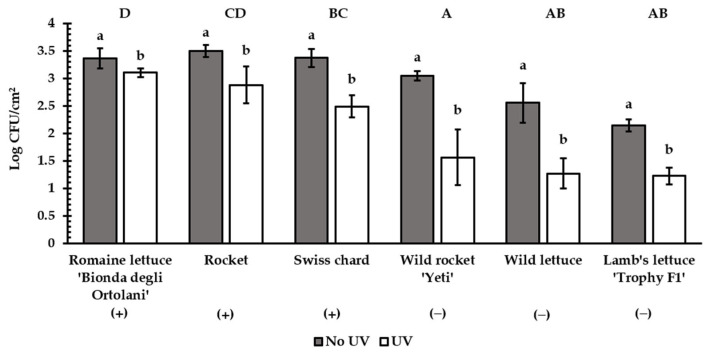
Reduction effect of UV treatment on *E. coli* ATCC 35218 attachment in the six selected accessions (+ = rougher; − = less rough). Different lowercase letters show significant differences (*p* < 0.05) for each accession; different uppercase letters show significant differences (*p* < 0.001) in the net bacterial population reduction between accessions (Tukey test, n = 5). Bars represent ± SD.

In the six selected accessions, *E. coli* ATCC 35218 survival was measured in the surface-inoculated leaves after UV treatment. UV rays significantly reduced *E. coli* ATCC 35218 contamination in all the accessions (Figure 5). Nevertheless, a lower reduction in the attachment was achieved in romaine lettuce ‘Bionda degli Ortolani’ (−8%) and rocket (−17%) compared to the accessions showing lower roughness (wild rocket ‘Yeti’, wild lettuce and lamb’s lettuce ‘Trophy F1′; −47% on average) (Figure 5). Swiss chard differed from ‘Bionda degli Ortolani’ and wild rocket ‘Yeti’.

## 4. Discussion

Intraspecific variability in the susceptibility to human pathogen contamination has been observed by many authors in several vegetable crops, among which lettuce was one of the most studied [34]. In particular, Jacob and Melotto [23] found differences in the attachment and persistence of *S. enterica* and *E. coli* between nine cultivars of this species. Our study confirmed differences in *E. coli* attachment within lettuces, even between accessions belonging to the same type (Table 2). Interestingly, ‘Bionda degli Ortolani’ and ‘Maraichere’, whose susceptibility resulted to be different, are both romaine lettuces. Romaine lettuce has been involved in many food disease outbreaks in the U.S. and, in particular, in *E. coli* infections [35,36,37,38]. These alerts are generically referred to this lettuce type, considered as one of the riskiest leafy vegetable in terms of foodborne diseases. However, since our data showed that romaine lettuce contamination varies by cultivar, a reduction in the risks associated with this lettuce could be achieved through varietal choice. Also, for *Diplotaxis tenuifolia*, we observed intraspecific variability (Table 2) which, to our knowledge, had not yet been found in this species. No differences in *E. coli* ATCC 35218 attachment were detected within the *Taraxacum campylodes*, *Brassica juncea, Brassica rapa*, *Cichorium intybus* and *Beta vulgaris* accessions. Conversely, the same accessions of *C. intybus* used in this study but compared at the microgreen stage (immature greens harvested when cotyledons the first pair of true leaves are more or less developed) showed a different susceptibility to *S. enterica* and *E. coli* contamination [39]. Actually, the stage of the leaf may affect the contamination, as noticed by Hunter [40].

It is known that the leaf micro-morphological traits influence the attachment and persistence of human pathogens on vegetables. Many studies have shown that stomata are involved in the contamination of leafy greens with *S. enterica* and *E. coli* [11,23,41,42]. Bacteria were found on guard cells, within and underneath the stomata cavity, and on the crevices in proximity to them [19]. In our study, the six selected baby leaf accessions differed in stomata (Table 3). Nevertheless, the Pearson’s test revealed that the bacterial retention (1.5 h after inoculation) and stomatal parameters were not correlated. This finding agrees with the results reported by Jacob and Melotto [23], who did not find a correlation between stomata aperture width, pore area, and density and *E. coli* or *S.* Typhimurium attachment onto leaves of different lettuce genotypes. On the other hand, the same authors found that the bacterial persistence 10 days after surface inoculation was correlated with the stomatal aperture width and pore area [23]. Also, Macarisin [11] observed that stomata density affected the persistence of *E. coli* O157:H7 in different spinach cultivars. Therefore, as suggested by Jacob and Melotto [23], for lettuce, stomata seem to have a role in bacterial penetration into the leaves, while the attachment might be influenced by other properties of the leaf surface. Traits such as trichomes, hydrophobicity, vein areas, cuticular waxes, surface irregularities and surface proteins and sugars can be associated with different attachment levels [40,43,44]. Palma-Salgado et al. [24] found that leaf surface roughness was positively correlated to *E. coli* adhesion in different leafy greens. Also, in spinach, roughness was an important factor determining the differential attachment and persistence of *E. coli* on leaves [11]. Our results support these previous findings. In fact, a significant correlation between the contamination level and roughness parameters (Ra and Rq values) was observed (Figure 4). 

Leaf roughness can also reduce the effectiveness of sanitizing treatments for vegetables. Doan et al. [14] and Yi et al. [45] reported that the variation in leaf topography accounted for differences in survival of *E. coli* in chlorine-treated leafy greens of different species and cultivars. Surface roughness revealed to be a factor protecting *E. coli* cells from treatment with chlorine also in fruits and seeds [46,47]. UV radiation can be used as an alternative to chemicals for the sanitization of food products [48]. Different studies demonstrated the effectiveness of UV rays to control foodborne pathogens in fresh-cut vegetables, including lettuce [49,50]. In general, the role of surface topography on the effectiveness of UV treatment for bacterial inactivation was demonstrated by Woodling and Moraro in stainless-steel surfaces [51]. In our study, UV treatment resulted in less *E. coli* ATCC 35218 attachment reduction in rougher accessions than in those showing less roughness (Figure 5). Therefore, our results suggest that leaf roughness may offer protective niches to bacteria from UV rays.

Finally, the positive correlation between *E. coli* ATCC 35218 attachment and leaf water content that we found could suggest that the internal water status may impact the bacterial contamination of the leaves. Interestingly, in Yadav et al. [25], leaf water content was positively correlated with bacterial colonization of the phyllosphere in eight Mediterranean plant species. *S. enterica* colonization was enhanced by water-congestion in green tomato fruits [52]. The mechanism by which tissue water amount influences bacterial contamination in plants could be related to physical and/or chemical factors associated to tissue turgidity.

## 5. Conclusions

The screening of 30 baby leaf accessions for the susceptibility to *E. coli* ATCC 35218 contamination confirmed that variability exists among different leafy crop species, types of the same species and cultivars. The differences within the same species suggest that the choice of the cultivar can be a means to reduce the risk of foodborne diseases linked to the consumption of salads. Even within var. *longifolia* of lettuce (romaine type), which is the most involved in food disease outbreaks in the U.S., and for this reason is considered one of the riskiest salads, it seems possible to find genotypes (e.g., cv. ‘Maraichere’) less susceptible to contamination than others (e.g., cv. ‘Bionda degli Ortolani’). These findings could find a practical application in orienting growers and the vegetable industry towards safer crops.

Our study also showed that leaf roughness and water content impact on *E. coli* ATCC 35218 attachment in baby greens. The use of an innovative portable 3D digital microscope highlighted the differences in the leaf roughness of six selected accessions. Among the measured leaf micro-morphological traits, roughness was the only one to be positively correlated with the *E. coli* ATCC 35218 contamination level. Roughness also somehow offered UV protection to bacteria. In fact, when *E. coli* ATCC 35218 retention in surface-inoculated leaves was measured after UV treatment, bacterial contamination was reduced to a minor extent in the rougher leaves. This finding further underlies the impact of leaf roughness on the safety of vegetables, suggesting that sanitization treatment through UV rays should be modulated accounting for this parameter. The correlation between *E. coli* ATCC 35218 attachment and leaf water content was also significant, suggesting that the internal water status can impact the bacterial contamination of the leaves.

Looking forward, new findings on leaf characteristics associated with lower susceptibility to attachment and proliferation of human pathogens could be used in selection breeding in order to obtain safer cultivars and thus increase food safety in the vegetable industry. In particular, our results identified roughness as a trait to be considered in breeding programs.

## Figures and Tables

**Table 1:** Detailed list of the 30 baby leaf accessions used in the study.

	Family	Specie	Variety/Type/Cultivar	Accession Name
1	Asteraceae	*Cichorium intybus* L.	var. *sativus* Witloof type	Witloof chicory
2			var. *foliosum* cv. ‘Magdeburgo’	Chicory ‘Magdeburgo’
3			-	Wild chicory Ingegnoli
4			-	Wild chicory B&T
5			-	Wild chicory local
6			var. *foliosum* cv. ‘Biondissima di Trieste’	Chicory ‘Biondissima di Trieste’
7			var. *foliosum* cv. ‘Spadona da taglio’	Chicory ‘Spadona da taglio’
8		*Cichorium endivia* L.	var. *crispum*	Endive
9		*Lactuca sativa* L.	var. *crispa* Lollo verde type	Lollo verde lettuce
10			var. *crispa* Lollo rossa type	Lollo rossa lettuce
11			var. *crispa* blonde type	Blonde lettuce
12			var. *crispa* cv. ‘Pamela’	Lettuce ‘Pamela’
13			var. *longifolia* cv. ‘Bionda degli ortolani’	Romaine lettuce ‘Bionda degli Ortolani’
14			var. *longifolia* cv. ‘Maraichere’	Romaine lettuce ‘Maraichere’
15		*Lactuca serriola* L.	-	Wild lettuce
16		*Taraxacum campylodes* G.E.Haglund	-	Dandelion local
17			-	Dandelion Ingegnoli
18	Brassicaceae	*Eruca sativa* Miller	-	Rocket
19		*Diplotaxis tenuifolia* L.	-	Wild rocket Ingegnoli
20			cv. ‘Yeti’	Wild rocket ‘Yeti’
21		*Brassica rapa* L.	subsp. *chinensis*	Pak choi
22			subsp. *nipposinica*	Mizuna
23		*Brassica juncea* L.	-	Wasabina leaf mustard
24			-	Red Giant leaf mustard
25			-	Red leaf mustard
26	Chenopodiaceae	*Beta vulgaris* L.	subsp. *cycla*	Swiss chard
27			subsp. *cycla* cv.‘Bull’s Blood Artica’	Red chard ‘Bull’s Blood Artica’
28		*Spinacia oleracea* L.	cv. ‘Cugoe RZ F1′	Spinach ‘Cugoe RZ F1′
29	Valerianeceae	*Valerianella locusta* L.	cv. ‘Trophy F1′	Lamb’s lettuce ‘Trophy F1′
30	Polygonaceae	*Rumex acetosa* L.	-	Sorrel

Seed source: Fratelli Ingegnoli, Milan, Italy (Accessions 1, 2, 3, 6, 7, 8, 13, 17, 19, 21, 29, 30); B&T World Seed, Aigues-Vives, France (Accession 4); Locally gathered in the wild in Florence, Italy (Accession 5); Sativa Bio, Rheinau, Switzerland (Accessions 9, 10, 22, 24); Maraldi Sementi, Cesena, Italy (Accessions 11, 12, 20, 26, 27, 28); Vivosem, Macerata, Italy (Accession 14); Provencemonamour, Paris, France (Accession 15); locally gathered in the wild in Lucca, Italy (Accession 16); Gargini sementi, Lucca, Italy (Accession 18); Tutto-Semi, Wilts, England (Accessions 23, 25).

**Table 2:** *E. coli* ATCC 35218 attachment in 30 accessions of baby leaves.

Accession	Attachment log CFU/cm^2^	Accession	Attachment log CFU/cm^2^
Romaine lettuce ‘Bionda degli Ortolani’	3.36 ± 0.36 a	Dandelion (local)	2.94 ± 0.32 abcd
Rocket	3.15 ± 0.14 ab	Chicory ‘Biondissima di Trieste’	2.93 ± 0.31 abcd
Swiss chard	3.11 ± 0.20 ab	Pak-choi	2.93 ± 0.24 abcd
Endive	3.11 ± 0.26 ab	Lollo verde lettuce	2.90 ± 0.30 abcd
Spinach ‘Cugoe RZ F1′	3.10 ± 0.23 ab	Blonde lettuce	2.87 ± 0.21 abcde
Mizuna	3.10 ± 0.20 ab	Red leaf mustard	2.83 ± 0.27 abcde
Wild chicory (B&T)	3.03 ± 0.43 abc	Wild chicory (Ingegnoli)	2.81 ± 0.36 abcde
Dandelion (Ingegnoli)	3.02 ± 0.46 abc	Red chard ‘Bull’s Blood Artica’	2.79 ± 0.27 abcde
Red Giant leaf mustard	3.01 ± 0.25 abc	Wild chicory (local)	2.68 ± 0.55 bcde
Wasabina leaf mustard	3.00 ± 0.48 abc	Romaine lettuce ‘Maraichere’	2.65 ± 0.34 bcde
Wild rocket (Ingegnoli)	2.98 ± 0.32 abcd	Lettuce ‘Pamela’	2.45 ± 0.20 cde
Chicory ‘Magdeburgo’	2.97 ± 0.46 abcd	Sorrel	2.40 ± 0.69 de
Lollo rossa lettuce	2.96 ± 0.50 abcd	Wild rocket ‘Yeti’	2.36 ± 0.52 e
Chicory ‘Spadona da taglio’	2.96 ± 0.21 abcd	Wild lettuce	2.34 ± 0.53 e
Witloof Chicory	2.95 ± 0.46 abcd	Lamb’s lettuce ‘Trophy F1′	1.68 ± 0.39 f

Different letters show significant differences for *p* ≤ 0.05, Tukey test. Data are means ± SD (n = 10).

**Table 3:** Stomatal parameters (density, length, width, rim area), roughness (Ra and Rq) of adaxial leaf surface and water content (%) in six selected accessions of baby leaves (+ = most susceptible to contamination; — = less susceptible).

Stomatal Density (n/mm^2^)	Stomata Length (µm)	Stomata Width (µm)	Stomatal Rim Area (µm^2^)	Ra	Rq	Water Content (%)
Romaine lettuce ‘Bionda degli Ortolani’ (+)						
71.96 ± 6.11 ab	25.93 ± 1.96 b	20.56 ± 3.28 c	23.03 ± 5.52 bc	58.81 ± 11.52 a	71.56 ± 13.71 a	95.36 ± 0.84 a
Rocket (+)						
92.20 ± 28.40 a	23.46 ± 1.39 c	15.66 ± 1.92 d	11.70 ± 3.55 c	45.81 ± 7.74 bc	56.58 ± 9.79 bc	93.66 ± 1.59 b
Swiss chard (+)						
61.65 ± 10.80 b	30.10 ± 1.98 a	21.85±3.38 b	45.58±6.78 a	54.70 ± 15.84 ab	66.92 ± 18.35 ab	92.66 ± 1.62 b
Wild rocket ‘Yeti’ (−)						
80.29 ± 25.67 ab	21.85 ± 1.64 d	14.38±2.23 e	24.35±7.63 b	41.33 ± 4.01 c	50.23 ± 5.01 c	92.08 ± 1.03 bc
Wild lettuce (−)						
-	-	-	-	39.43 ± 12.42 c	51.14 ± 15.49 c	89.79 ± 1.13 c
Lamb’s lettuce ‘Trophy F1′ (−)						
57.14 ± 5.47 b	30.19 ± 1.33 a	23.83 ± 2.05 a	19.84 ± 1.52 bc	24.74 ± 10.92 d	31.38 ± 14.24 d	90.22 ± 2.30 c

Different letters show significant differences for *p* ≤ 0.05, Tukey test. Data are means ± SD (n = 3–152).

## Data Availability

The data presented in this study are available on request from the corresponding author.

## Supplementary Materials

The following supporting information can be downloaded at: https://www.mdpi.com/article/10.3390/biology12010102/s1, Table S1: Number of leaves (NL), leaf fresh weight (FW) and dry weight (DW), leaf area (LA); and leaf water content (LWC) of the 30 baby leaf accessions (average ± SD); Table S2: Colour (a*, b*, L*) of the 30 baby leaf accessions (average ± SD); Video S1: 3D reconstruction of the surface under inspection obtained elaborating a sequence of defocused images implementing a suitable algorithm based on shape from the focusing technique.

## References

[1] Di Gioia F., Renna M., Santamaria P. (2017). Sprouts, microgreens and “baby leaf” vegetables. Minimally Processed Refrigerated Fruits and Vegetables.

[2] Sucheta Singla G., Chaturvedi K., Sandhu P.P., Siddiqui M.W. (2020). Status and recent trends in fresh-cut fruits and vegetables. Fresh-Cut Fruits and Vegetables.

[3] Grand View Research (2021). Packaged Salad Market Size, Share & Trends Analysis Report by Product (Vegetarian, Non-Vegetarian), by Processing (Organic, Conventional), by Type, by Distribution Channel, by Region, and Segment Forecasts, 2021–2028. San Francisco (CA). https://www.grandviewresearch.com/industry-analysis/packaged-salad-market.

[4] Lenzi A., Orlandini A., Bulgari R., Ferrante A., Bruschi P. (2019). Antioxidant and mineral composition of three wild leafy species: A comparison between microgreens and baby greens. Foods.

[5] Bartz J.A., Marvasi M., Teplitski M., Matthews K.R., Sapers G.M., Gerba C.P. (2014). Salmonella and tomatoes. The Produce Contamination Problem: Causes and Solution.

[6] Mir S.A., Shah M.A., Mir M.M., Dar B., Greiner R., Roohinejad S. (2018). Microbiological contamination of ready-to-eat vegetable salads in developing countries and potential solutions in the supply chain to control microbial pathogens. Food Control.

[7] Ölmez H., Kotzekidou P. (2016). Foodborne pathogenic bacteria in fresh-cut vegetables and fruits. Food Hygiene and Toxicology in Ready-to-Eat Foods.

[8] Centers for Disease Control and Prevention (2022). *E. coli* Outbreak Linked to Packaged Salads. https://www.cdc.gov/ecoli/2021/o157h7-12-21/index.html.

[9] Dunne W.M. (2002). Bacterial adhesion: Seen any good biofilms lately?. Clin. Microbiol. Rev..

[10] Grivokostopoulos N.C., Makariti I.P., Hilaj N., Apostolidou Z., Skandamis P.N. (2022). Internalization of *Salmonella* in leafy greens and the impact on acid tolerance. Appl. Environ. Microbiol..

[11] Macarisin D., Patel J., Bauchan G., Giron J.A., Ravishankar S. (2013). Effect of spinach cultivar and bacterial adherence factors on survival of *Escherichia coli* O157:H7 on spinach leaves. J. Food Prot..

[12] Sela S., Manulis-Sasson S. (2015). What else can we do to mitigate contamination of fresh produce by foodborne pathogens?. Microb. Biotechnol..

[13] Luna-Guevara J.J., Arenas-Hernandez M.M., Martínez de la Peña C., Silva J.L., Luna-Guevara M.L. (2019). The role of pathogenic *E. coli* in fresh vegetables: Behavior, contamination factors, and preventive measures. Int. J. Microb..

[14] Doan H.K., Antequera-Gómez M.L., Parikh A.N., Leveau J.H.J. (2020). Leaf surface topography contributes to the ability of *Escherichia coli* on leafy greens to resist removal by washing, escape disinfection with chlorine, and disperse through splash. Front. Microbiol..

[15] Doan H.K., Ngassam V.N., Gilmore S.F., Tecon R., Parikh A.N., Leveau J.H.J. (2020). Topography-driven shape, spread, and retention of leaf surface water impacts microbial dispersion and activity in the phyllosphere. Phytobiomes J..

[16] Melotto M., Underwood W., He S.Y. (2008). Role of stomata in plant innate immunity and foliar bacterial diseases. Annu. Rev. Phytopathol..

[17] Yaron S., Römling U. (2014). Biofilm formation by enteric pathogens and its role in plant colonization and persistence. Microb. Biotechnol..

[18] Kroupitski Y., Pinto R., Brandl M.T., Belausov E., Sela S. (2009). Interactions of *Salmonella enterica* with lettuce leaves. J. Appl. Microbiol..

[19] Saldana Z., Sánchez E., Xicohtencatl-Cortes J., Puente J.L., Girón J.A. (2011). Surface structure involved in plant stomata and leaf colonization by Shiga-toxigenic *Escherichia coli* O157:H7. Front. Microbiol..

[20] Wang H., Zhou B., Feng H., Gómez-López V.M. (2012). Surface characteristics of fresh produce and their impact on attachment and removal of human pathogens on produce surfaces. Decontamination of Fresh and Minimally Processed Produce.

[21] Melotto M., Panchal S., Roy D. (2014). Plant innate immunity against human bacterial pathogens. Front. Microbiol..

[22] Mitra R., Cuesta-Alonso E., Wayadande A.C., Talley J., Gilliland S., Fletcher J. (2009). Effect of route of introduction and host cultivar on the colonization, internalization, and movement of the human pathogen *Escherichia coli* O157:H7 in spinach. J. Food Prot..

[23] Jacob C., Melotto M. (2020). Human pathogen colonization of lettuce dependent upon plant genotype and defense response activation. Front. Plant Sci..

[24] Palma-Salgado S., Ku K.-M., Dong M., Nguyen T.H., Juvik J.A., Feng H. (2020). Adhesion and removal of *E. coli* K12 as affected by leafy green produce epicuticular wax composition, surface roughness, produce and bacterial surface hydrophobicity, and sanitizers. Int. J. Food Microbiol..

[25] Yadav R.K.P., Karamanoli K., Vokou D. (2005). Bacterial colonization of the phyllosphere of Mediterranean perennial species as influenced by leaf structural and chemical features. Microb. Ecol..

[26] Chapman T.A., Wu X.Y., Barchia I., Bettelheim K.A., Driesen S., Trott D., Wilson M., Chin J.J.C. (2006). Comparison of virulence gene profiles of *Escherichia coli* strains isolated from healthy and diarrheic swine. Appl. Environ. Microbiol..

[27] Marvasi M., Noel J.T., George A.S., Farias M.A., Jenkins K.T., Hochmuth G., Xu Y., Giovanonni J.J., Teplitski M. (2014). Ethylene signalling affects susceptibility of tomatoes to *Salmonella*. Microb. Biotechnol..

[28] Pathan A., Bond J., Gaskin R. (2010). Sample preparation for SEM of plant surfaces. Mater. Today.

[29] Rasband W.S. (1997–2016). ImageJ.

[30] Cacciari I., Ciofini D., Mascalchi M., Mencaglia A., Siano S. (2012). Novel approach to the microscopic inspection during laser cleaning treatments of artworks. Anal. Bioanal. Chem..

[31] Siano S., Mencaglia A.A., Cacciari I. (2017). Microscopy Optoelectric Device with Focus Scanning. U.S. Patent.

[32] Cacciari I., Mencaglia A.A., Siano S. (2013). Micromorphology of gold jewels: A novel algorithm for 3D reconstruction and its quality assessment. Optics for Arts, Architecture, and Archaeology IV, Proceedings of the SPIE Optical Metrology.

[33] (2021). Standard—Geometrical Product Specifications (GPS)—Surface Texture: Areal—Part 2: Terms, Definitions and Surface Texture Parameters.

[34] Lenzi A., Marvasi M., Baldi A. (2020). Agronomic practices to limit pre-and post-harvest contamination and proliferation of human pathogenic Enterobacteriaceae in vegetable produce. Food Control.

[35] Slayton R., Turabelidze G., Bennett S., Schwensohn C., Yaffee A., Khan F., Butler C., Trees E., Ayers T., Davis M. (2013). Outbreak of Shiga toxin-producing *Escherichia coli* (STEC) O157:H7 associated with romaine lettuce consumption, 2011. PLoS ONE.

[36] Taylor E.V., Nguyen T.A., Machesky K.D., Koch E., Sotir M.J., Bohm S.R., Folster P., Bokanyi R., Kupper A., Bidol S.A. (2013). Multistate outbreak of *Escherichia coli* O145 infections associated with romaine lettuce consumption, 2010. J. Food Prot..

[37] Coulombe G., Catford A., Martinez-Perez A., Buenaventura E. (2020). Outbreaks of *Escherichia coli* O157:H7 infections linked to romaine lettuce in Canada from 2008 to 2018: An Analysis of Food Safety Context. J. Food Prot..

[38] Waltenburg M.A., Schwensohn C., Madad A., Seelman S.L., Peralta V., Koske S.E., Boyle M.M., Arends K., Patel K., Mattioli M. (2022). Two multistate outbreaks of a reoccurring Shiga toxin-producing *Escherichia coli* strain associated with romaine lettuce: USA, 2018–2019. Epidemiol. Infect..

[39] Lenzi A., Baldi A., Lombardelli L., Truschi S., Marvasi M., Bruschi P. (2022). Contamination of microgreens by *Salmonella enterica* and *Escherichia coli* is influenced by selection breeding in chicory (*Cichorium intybus* L.). Food Qual. Saf..

[40] Hunter P.J., Shaw R.K., Berger C.N., Frankel G., Pink D., Hand P. (2015). Older leaves of lettuce (*Lactuca* spp.) support higher levels of *Salmonella enterica* ser. Senftenberg attachment and show greater variation between plant accessions than do younger leaves. FEMS Microbiol. Lett..

[41] Gomes C., Da Silva P., Moreira R.G., Castell-Perez E., Ellis E.A., Pendleton M. (2009). Understanding *E. coli* internalization in lettuce leaves for optimization of irradiation treatment. Int. J. Food Microbiol..

[42] Golberg D., Kroupitski Y., Belausov E., Pinto R., Sela S. (2011). *Salmonella* Typhimurium internalization is variable in leafy vegetables and fresh herbs. Int. J. Food Microbiol..

[43] Brandl M.T., Cox C.E., Teplitski M. (2013). *Salmonella* interactions with plants and their associated microbiota. Phytopathology.

[44] Ku K.-M., Chiu Y.-C., Shen C., Jenks M. (2020). Leaf cuticular waxes of lettuce are associated with reduced attachment of the foodborne pathogen *Salmonella* spp. at harvest and after postharvest storage. LWT.

[45] Yi J., Leveau J.H., Nitin N. (2022). Role of multiscale leaf surface topography in antimicrobial efficacy of chlorine-based sanitizers. J. Food Eng..

[46] Wang H., Feng H., Liang W., Luo Y., Malyarchuk V. (2009). Effect of surface roughness on retention and removal of *Escherichia coli* O157:H7 on surfaces of selected fruits. J. Food Sci..

[47] Fransisca L., Feng H. (2012). Effect of surface roughness on inactivation of *Escherichia coli* O157:H7 87-23 by new organic acid–surfactant combinations on alfalfa, broccoli, and radish seeds. J. Food Prot..

[48] Singh H., Bhardwaj S.K., Khatri M., Kim K.-H., Bhardwaj N. (2021). UVC radiation for food safety: An emerging technology for the microbial disinfection of food products: A Review. Chem. Eng. J..

[49] Chun H.H., Kim J.Y., Song K.B. (2010). Inactivation of foodborne pathogens in ready-to-eat salad using UV-C irradiation. Food Sci. Biotechnol..

[50] Kim Y.H., Jeong S.G., Back K.H., Park K.H., Chung M.S., Kang D.H. (2013). Effect of various conditions on inactivation of *Escherichia coli* O157:H7, *Salmonella typhimurium*, and Listeria monocytogenes in fresh-cut lettuce using ultraviolet radiation. Int. J. Food Microbiol..

[51] Woodling S.E., Moraru C.I. (2005). Influence of surface topography on the effectiveness of pulsed light treatment for the inactivation of *Listeria innocua* on stainless-steel surfaces. J. Food Sci..

[52] Marvasi M., Hochmuth G.J., Giurcanu M.C., George A.S., Noel J.T., Bartz J., Teplitski M. (2013). Factors that affect proliferation of *Salmonella* in tomatoes post-harvest: The roles of seasonal effects, irrigation regime, crop and pathogen genotype. PLoS ONE.

