# Supplementary material for: Foliar Roughness and Water Content Impact on Escherichia coli Attachment in Baby Leafy Greens
Source: Biology (Basel). 2023 Jan 9;12(1):102. doi: 10.3390/biology12010102 (PMC9855646; doi:10.3390/biology12010102)
Supplement: Supplementary file 1 [file biology-12-00102-s001.zip › Supplementary materials.pdf]

**Table S1.** Number of leaves (NL), leaf fresh weight (FW) and dry weight (DW), leaf area (LA); and leaf water content (LWC) of the 30 baby leaf accessions (average ± SD).

| Accession | NL | FW (g) | DW (g) | LA ($cm^2$) | LWC (%) |
|---|---|---|---|---|---|
| Witloof chicory | 6.08 ± 0.79 | 3.67 ± 1.69 | 0.28 ± 0.13 | 130.88 ± 45.21 | 92.53 ± 1.21 |
| Chicory ‘Magdeburgo’ | 4.50 ± 0.52 | 2.20 ± 0.51 | 0.17 ± 0.05 | 85.15 ± 19.62 | 92.61 ± 1.07 |
| Wild chicory (Ingegnoli) | 6.25 ± 0.87 | 1.69 ± 0.51 | 0.13 ± 0.04 | 63.58 ± 14.63 | 92.12 ± 2.08 |
| Wild chicory (B&T) | 5.83 ± 0.72 | 1.76 ± 0.77 | 0.16 ± 0.06 | 64.73 ± 22.16 | 90.98 ± 2.00 |
| Wild chicory (Local) | 6.08 ± 1.00 | 1.10 ± 0.50 | 0.10 ± 0.04 | 47.64 ± 20.41 | 90.48 ± 3.88 |
| Chicory ‘Biondissima di Trieste’ | 5.25 ± 1.06 | 2.28 ± 0.80 | 0.16 ± 0.07 | 105.44 ± 36.59 | 93.08 ± 0.91 |
| Chicory ‘Spadona da Taglio’ | 5.42 ± 1.16 | 2.68 ± 0.98 | 0.24 ± 0.08 | 91.69 ± 33.24 | 90.63 ± 2.96 |
| Endive | 6.75 ± 1.06 | 1.48 ± 0.42 | 0.11 ± 0.03 | 46.10 ± 14.21 | 92.32 ± 2.60 |
| Lollo verde lettuce | 5.50 ± 0.90 | 3.67 ± 0.79 | 0.17 ± 0.05 | 136.82 ± 31.03 | 95.58 ± 0.57 |
| Lollo rossa lettuce | 5.42 ± 1.16 | 3.37 ± 1.25 | 0.17 ± 0.08 | 133.62 ± 44.17 | 94.96 ± 0.77 |
| Blonde lettuce | 7.00 ± 0.74 | 3.59 ± 1.03 | 0.14 ± 0.04 | 121.13 ± 26.69 | 95.97 ± 0.65 |
| Lettuce ‘Pamela’ | 6.83 ± 1.11 | 3.40 ± 1.32 | 0.20 ± 0.10 | 144.41 ± 48.07 | 94.11 ± 1.05 |
| Romaine lettuce ‘Bionda degli Ortolani’ | 6.92 ± 1.00 | 3.21 ± 0.86 | 0.15 ± 0.05 | 97.73 ± 25.67 | 95.40 ± 0.81 |
| Romaine lettuce ‘Maraichere’ | 7.08 ± 1.08 | 2.73 ± 1.31 | 0.16 ± 0.08 | 82.84 ± 34.90 | 93.77 ± 1.86 |
| Wild lettuce | 7.75 ± 0.96 | 1.52 ± 0.65 | 0.16 ± 0.09 | 66.11 ± 23.82 | 89.79 ± 1.13 |
| Dandelion (Local) | 5.27 ± 0.79 | 0.97 ± 0.38 | 0.12 ± 0.03 | 49.66 ± 18.37 | 86.09 ± 7.21 |
| Dandelion (Ingegnoli) | 5.82 ± 1.54 | 1.26 ± 0.55 | 0.14 ± 0.06 | 67.50 ± 24.15 | 87.48 ± 5.97 |
| Rocket | 6.67 ± 1.23 | 3.18 ± 1.35 | 0.21 ± 0.12 | 86.76 ± 32.32 | 93.66 ± 1.59 |
| Wild rocket | 7.92 ± 0.67 | 1.51 ± 0.46 | 0.10 ± 0.02 | 36.62 ± 10.82 | 93.12 ± 1.11 |
| Wild rocket ‘Yeti’ | 7.00 ± 0.76 | 1.13 ± 0.66 | 0.09 ± 0.04 | 28.58 ± 14.05 | 92.08 ± 1.03 |
| Pak choi | 6.50 ± 1.17 | 5.09 ± 1.49 | 0.21 ± 0.07 | 91.05 ± 21.79 | 95.91 ± 0.58 |
| Mizuna | 7.58 ± 1.51 | 3.03 ± 1.53 | 0.20 ± 0.12 | 63.51 ± 26.47 | 93.69 ± 1.12 |
| Wasabina leaf mustard | 7.25 ± 1.06 | 3.24 ± 1.55 | 0.24 ± 0.11 | 85.54 ± 32.95 | 92.31 ± 1.68 |
| Red Giant leaf mustard | 6.67 ± 0.78 | 7.26 ± 4.04 | 0.41 ± 0.24 | 155.40 ± 68.62 | 94.36 ± 0.76 |
| Red leaf mustard | 6.58 ± 0.90 | 6.31 ± 3.82 | 0.38 ± 0.23 | 145.90 ± 79.81 | 93.94 ± 0.47 |
| Swiss chard | 5.67 ± 1.15 | 2.77 ± 1.35 | 0.19 ± 0.08 | 73.28 ± 35.74 | 92.66 ± 1.62 |
| Swiss chard 'Bull's Blood Artica' | 5.08 ± 0.90 | 0.79 ± 0.31 | 0.04 ± 0.02 | 19.11 ± 7.55 | 94.23 ± 2.46 |
| Spinach | 6.17 ± 0.83 | 2.71 ± 1.10 | 0.18 ± 0.07 | 59.38 ± 21.18 | 93.20 ± 0.42 |
| Lamb’s lettuce ‘Trophy F1’ | 7.83 ± 0.39 | 0.48 ± 0.10 | 0.05 ± 0.01 | 21.70 ± 4.38 | 90.22 ± 2.30 |
| Sorrel | 4.83 ± 0.72 | 2.34 ± 0.73 | 0.16 ± 0.07 | 67.00 ± 18.72 | 93.20 ± 1.12 |

**Table S2.** Colour (a*, b*, L*) of the 30 baby leaf accessions (average ± SD).

| **Accession** | **a*** | **b*** | **L*** |
|---|---|---|---|
| Witloof chicory | -10,22 ± 0,29 | 31,28 ± 2,91 | 47,10 ± 1,65 |
| Chicory ‘Magdeburgo’ | -7,93 ± 0,93 | 26,52 ± 2,63 | 43,95 ± 1,66 |
| Wild chicory (Ingegnoli) | -8,79 ± 0,42 | 23,33 ± 2,02 | 42,11 ± 0,68 |
| Wild chicory (B&T) | -7,74 ± 0,55 | 27,26 ± 3,70 | 41,74 ± 1,80 |
| Wild chicory (Local) | -7,30 ± 1,17 | 22,04 ± 2,26 | 39,38 ± 2,25 |
| Chicory ‘Biondissima di Trieste’ | -10,46 ± 0,77 | 37,05 ± 3,31 | 50,82 ± 2,01 |
| Chicory ‘Spadona da taglio’ | -7,97 ± 1,15 | 27,52 ± 2,94 | 42,95 ± 2,13 |
| Endive | -15,67 ± 2,77 | 43,78 ± 8,35 | 65,34 ± 2,86 |
| Lollo verde lettuce | -14,39 ± 0,93 | 46,25 ± 4,06 | 66,04 ± 3,56 |
| Lollo rossa lettuce | -11,88 ± 4,19 | 49,27 ± 1,37 | 60,04 ± 4,67 |
| Blonde lettuce | -14,14 ± 2,84 | 49,87 ± 1,21 | 61,54 ± 3,04 |
| Lettuce ‘Pamela’ | -6,57 ± 2,56 | 41,60 ± 2,80 | 52,91 ± 2,52 |
| Romaine lettuce ‘Bionda degli Ortolani’ | -11,62 ± 2,07 | 42,45 ± 3,61 | 54,57 ± 3,67 |
| Romaine lettuce ‘Maraichere’ | -6,34 ± 0,59 | 29,02 ± 3,02 | 41,67 ± 2,50 |
| Wild lettuce | -3,40 ± 1,31 | 29,61 ± 2,69 | 38,03 ± 1,03 |
| Dandelion (Local) | -7,98 ± 0,56 | 26,73 ± 0,48 | 42,23 ± 0,10 |
| Dandelion (Ingegnoli) | -8,84 ± 1,80 | 33,28 ± 3,40 | 53,65 ± 3,11 |
| Rocket | -3,23 ± 1,41 | 31,53 ± 3,19 | 39,59 ± 2,23 |
| Wild rocket | -8,34 ± 1,78 | 24,65 ± 4,06 | 41,38 ± 1,52 |
| Wild rocket ‘Yeti’ | -5,88 ± 1,72 | 31,19 ± 3,26 | 42,23 ± 2,74 |
| Pak choi | -6,02 ± 0,37 | 30,57 ± 3,40 | 41,53 ± 3,24 |
| Mizuna | -5,64 ± 1,27 | 33,83 ± 1,23 | 44,39 ± 2,06 |
| Wasabina leaf mustard | -6,13 ± 1,82 | 22,85 ± 4,03 | 34,59 ± 2,80 |
| Red Giant leaf mustard | -5,10 ± 1,73 | 30,67 ± 2,71 | 41,48 ± 0,99 |
| Red leaf mustard | -1,15 ± 0,69 | 37,83 ± 0,69 | 44,19 ± 0,86 |
| Swiss chard | -7,53 ± 0,53 | 29,36 ± 1,32 | 42,43 ± 1,14 |
| Swiss chard 'Bull's Blood Artica' | 12,71 ± 2,11 | -2,96 ± 0,37 | 22,89 ± 2,07 |
| Spinach | -8,61 ± 0,20 | 17,14 ± 2,17 | 36,26 ± 0,72 |
| Lamb’s lettuce ‘Trophy F1’ | -7,19 ± 1,23 | 24,85 ± 3,53 | 39,27 ± 3,46 |
| Sorrel | -8,74 ± 0,67 | 23,03 ± 1,69 | 40,80 ± 2,27 |
